# Mechanisms Underlying Male Reproductive Toxicity Induced by Sublethal β-Cypermethrin Exposure in *Antheraea pernyi* (Guérin-Méneville, 1855) (Saturniidae)

**DOI:** 10.3390/insects17060633

**Published:** 2026-06-15

**Authors:** Xin Chen, Tianyi Zhang, Liang Xu, Junshan Chen, Peifeng Liu, Fengquan Liu, Shiwen Zhao, Miaomiao Chen, Xisheng Li

**Affiliations:** Sericultural Research Institute of Liaoning Province, Dandong 118100, China; chenxinzk@163.com (X.C.); zhtiayi@163.com (T.Z.); xulianglcs@126.com (L.X.); chenjunshan2026@163.com (J.C.); liupeifeng2026@163.com (P.L.); liufengquan1028@163.com (F.L.); zsw051199@163.com (S.Z.); miaozl8887@163.com (M.C.)

**Keywords:** *Antheraea pernyi*, β-cypermethrin, male reproductive toxicity, spermatogenesis, sperm dysfunction, dual-hit mechanism

## Abstract

Pyrethroid insecticides are widely used in agriculture, but their sublethal effects on male reproduction in *Antheraea pernyi* (Guérin-Méneville, 1855) (Saturniidae) remain largely unknown. Here, we evaluated the effects of sublethal β-cypermethrin exposure (LC_20_ = 0.0074 mg/L) on gonadal development, testicular morphology, sperm quantity and function, mating behavior, and post-mating egg traits from the larval to adult stages and investigated the molecular mechanisms using transcriptome sequencing. Insecticide stress impaired testicular development and disrupted spermatogenesis, leading to a 25% reduction in eupyrene sperm bundles, a 10.72% decrease in acrosin activity, and a 16.75% decrease in the curvilinear velocity of apyrene sperm. Mating willingness decreased by 17.78 percentage points, and females mated with treated males produced 20.45% fewer eggs. Transcriptome analysis identified 1193 differentially expressed genes (|log_2_FC| > 1, FDR < 0.01). Detoxification and stress response genes (e.g., *CYP3A27*, *GSTD1*, *ABCB1*, and *Hsp70*) were predominantly up-regulated, while reproduction-related genes (e.g., *dnal1*, *tubb1*, *ATPsynbeta*, *far1*, *JHAMT*, *mei-41*, and *dna2*) were down-regulated. This study demonstrates that sublethal β-cypermethrin exposure induces male reproductive toxicity in *A. pernyi* through a “dual-hit” mechanism: persistently activating the detoxification system (leading to energy depletion) while directly suppressing reproductive genes, ultimately resulting in impaired germ cell development, defective sperm function, and reduced fertility.

## Simple Summary

The Chinese oak silkworm, *Antheraea pernyi*, is an economically important insect reared in the wild in China, contributing significantly to agriculture. However, pyrethroid insecticides widely used on crops may harm the reproduction of nontarget insects like this silkworm. In this study, we explored how a low, non-lethal dose of the insecticide β-cypermethrin affects male reproduction and the underlying mechanisms. Our results showed that the insecticide damaged testis development and sperm production, leading to fewer and abnormally shaped sperm, as well as reduced sperm function. It also decreased male mating willingness, which in turn lowered the number of eggs laid by females and the hatching rate of those eggs. By transcriptome analysis, we found that the insecticide up-regulated genes involved in detoxification and stress response while down-regulating genes crucial for reproduction, including those related to cytoskeletal structure, energy metabolism, lipid metabolism, hormonal regulation, and DNA damage response and repair. This imbalance between “defense activation” and “reproductive inhibition” reveals a complex mechanism by which pesticides cause lasting harm to insect reproduction.

## 1. Introduction

*Antheraea pernyi* (Guérin-Méneville, 1855) (Saturniidae) is an economically important insect species reared in the wild in China. Its silk is highly valued for producing high-grade textiles and specialized military materials due to its soft luster, moisture absorption, breathability, and strength [1]. Additionally, the pupae are rich in high-quality protein, unsaturated fatty acids, vitamins, and minerals, making them a popular green food source and an important protein supplement in aquaculture and livestock farming [2].

Other byproducts, such as adult moths and silkworm feces, also show considerable potential for development in the pharmaceutical and health product industries [1]. Currently, the *A. pernyi* industry has formed a complete industrial chain integrating oak tree cultivation, silkworm rearing, cocoon reeling, pupa processing, and byproduct utilization, serving as a pillar industry in major silk-producing regions [3].

Due to its outdoor rearing system, *A. pernyi* is inevitably exposed to pesticides applied to adjacent agricultural fields. In recent years, adjustments in agricultural planting structures and the expansion of chemical control areas, coupled with the widespread use of modern plant protection equipment such as unmanned aerial vehicles (UAVs), have significantly increased the risk of pesticide drift [4,5].

Poisoning incidents in *A. pernyi* have become increasingly frequent, causing substantial economic losses to farmers and severely constraining the sustainable development of the industry [6,7,8,9]. Among the various pesticides used, β-cypermethrin, a pyrethroid insecticide, warrants particular attention regarding its toxic effects on this nontarget insect. In the Dandong region (Liaoning Province), it is widely applied to corn, apple, pear, cabbage, and tomato for pest control.

β-cypermethrin is a representative second-generation pyrethroid insecticide that exerts its insecticidal activity by acting on voltage-gated sodium channels, thereby disrupting normal nervous system function [10]. Due to its high efficacy, broad-spectrum activity, low mammalian toxicity, and rapid degradation in the environment, it has become one of the most commonly used insecticides for controlling lepidopteran, coleopteran, and hemipteran pests in global agriculture [11]. However, its large-scale and continuous use has raised concerns about pesticide residues entering nontarget habitats through spray drift, surface runoff, and soil leaching.

Numerous studies have shown that the effects of pesticides on nontarget insects extend beyond acute lethal toxicity; sublethal effects—defined as adverse effects occurring at concentrations below the direct lethal threshold—deserve particular attention, as they can induce suboptimal physiological states in terms of behavior, development, and reproduction [12,13]. Such effects are often not immediately observable but may have profound impacts on population dynamics through cumulative damage.

The male reproductive system, being highly sensitive to environmental stress, has become an important endpoint for assessing the sublethal toxicity of pollutants [14,15]. In lepidopteran insects, male reproductive development is a highly ordered and energy-intensive biological process. From mitotic proliferation of spermatogonia and meiotic division of spermatocytes during the larval stage, to spermatid metamorphosis during the pupal stage, and finally to sperm maturation and functional acquisition in adults, each stage is precisely regulated by coordinated gene expression networks and hormonal signaling pathways [16,17].

*A. pernyi* reproductive development exhibits two distinct characteristics: first, testicular development is tightly coupled with larval–pupal–adult metamorphosis, meaning that environmental stress experienced during the larval stage can persist into the adult stage [18]; second, *A. pernyi* produces two types of sperm—eupyrene sperm, which are responsible for fertilization and genetic transmission, and apyrene sperm, which, although not involved in fertilization, facilitate the transport and activation of eupyrene sperm within the female spermatheca through their motility [19,20].

Therefore, a comprehensive assessment of male reproductive toxicity requires a multi-tiered evaluation system encompassing gonadal development, gametogenesis, sperm morphology and function, individual mating behavior, and population-level reproductive output [15].

Current research on the reproductive toxicity of pyrethroid insecticides in nontarget insects has primarily focused on model species such as *Drosophila melanogaster* (Meigen, 1830) (Drosophilidae) and *Apis mellifera* (Linnaeus, 1758) (Apidae). Sublethal exposure to β-cypermethrin has been shown to impair spermatogenesis, disrupt testicular structure, and reduce mating success in *D. melanogaster* [21]. In *A. mellifera*, it induces decreased semen quality, reduced sperm motility, and diminished egg-laying capacity in queens [22,23].

However, studies on the reproductive toxicity of these insecticides in economically important insects such as *A. pernyi* remain scarce. More importantly, existing studies have largely focused on physiological phenotypic descriptions, with limited systematic investigation into the molecular mechanisms by which sublethal pesticide stress induces reproductive damage. In particular, whether the detoxification system activated by insects in response to pesticide stress imposes a “metabolic cost” on reproductive development, and whether persistent up-regulation of detoxification genes interferes with endogenous hormone metabolism and energy allocation, remains poorly understood.

Against this background, the present study used Liaocanda 9, a major *A. pernyi* strain, to determine the sublethal concentration (LC_20_ = 0.0074 mg/L) of β-cypermethrin in fifth-instar male larvae through laboratory bioassays. The effects of sublethal exposure on testicular development, testicular morphology, sperm morphology and function, mating behavior, and offspring hatching rate were systematically evaluated.

Concurrently, transcriptome sequencing was employed to analyze the molecular response network in the testis, and key differentially expressed genes were identified through functional annotation and pathway enrichment analysis, followed by RT-qPCR validation. The aim was to elucidate the molecular mechanisms underlying male reproductive toxicity induced by sublethal β-cypermethrin exposure and to reveal the intrinsic relationship between detoxification system activation and reproductive impairment.

The findings are expected to provide theoretical guidance for the rational use of pesticides in *A. pernyi* farming regions, offer new insights into the evolutionary trade-off between detoxification and reproduction in insects, and contribute to a comprehensive assessment of the ecological risks posed by pyrethroid insecticides.

## 2. Materials and Methods

### 2.1. Experimental Insects

*A. pernyi* larvae used in this study were the Liaocanda 9 strain, which was provided by the Breeding Research Office of the Liaoning Provincial Sericultural Research Institute. Larvae were reared in the wild at a research base free from pesticide contamination until the fourth instar during the spring, with strict measures taken to ensure no pesticide exposure during rearing. Healthy male larvae of uniform developmental stage were selected and transferred to a climate-controlled chamber. Insecticide exposure experiments were conducted on the first day after molting into the fifth instar.

### 2.2. Insecticides and Chemicals

β-Cypermethrin (99%) {International Union of Pure and Applied Chemistry (IUPAC) name: (RS)-α-cyano-3-phenoxybenzyl (1R, 3R)-3-(2, 2-dichloroethenyl)-2, 2-dimethyl cyclopropanecarboxylate} was purchased from Yuanye Biotechnology Co., Ltd. (Shanghai, China).

The sperm acrosin activity assay kit (substrate enzyme method) was purchased from Ruiaijin Biotechnology Co., Ltd. (Tianjin, China). Grace’s insect cell culture medium was obtained from Procell Life Science & Technology Co., Ltd. (Wuhan, China). Nuclease-free water and phosphate-buffered saline (PBS) were purchased from Solarbio Science & Technology Co., Ltd. (Beijing, China).

### 2.3. Toxicological Bioassays

To determine the toxicity of β-cypermethrin to male larvae of the Liaocanda 9 strain, a modified leaf-dipping bioassay method was employed [24]. Briefly, the technical-grade β-cypermethrin was completely dissolved in acetone to prepare a stock solution, which was then diluted with distilled water to five concentrations: 0.008, 0.016, 0.032, 0.064, and 0.128 mg/L.

Fresh oak branches were trimmed to approximately 40 cm in length and immersed in each test solution concentration for 20 s. After immersion, the branches were air-dried under ambient conditions at 25 ± 1 °C and 50 ± 5% relative humidity. Branches treated with an equivalent volume of acetone served as the vehicle control. The treated branches were placed upright in transparent plastic cups (11 cm in diameter, 14 cm in height) with the base inserted into water (approximately two-thirds of the cup volume) to maintain freshness.

Fifth-instar larvae on the first day after molting (fasted before the bioassay) were placed onto the branches at a density of 20 larvae per concentration, with three biological replicates. All larvae were reared in a climate-controlled chamber under the following conditions: temperature 25 ± 1 °C, relative humidity 75 ± 5%, and a photoperiod of 16 h light: 8 h dark. Mortality was recorded 48 h after treatment.

### 2.4. Sublethal Exposure to β-Cypermethrin

Based on the results of the toxicity bioassay, the LC_20_ = 0.0074 mg/L (Table S1) of β-cypermethrin was selected for subsequent sublethal effect studies. The experiment consisted of a treatment group and a control group, each with three biological replicates, and each replicate comprised 120 1-day-old fifth-instar male larvae. Larvae in the treatment group were exposed to β-cypermethrin using the method described in Section 2.3 for 48 h, while larvae in the control group were subjected to the same procedure using acetone-treated branches.

After the 48 h exposure period, surviving larvae were transferred to fresh, uncontaminated oak branches and reared under controlled conditions (temperature 25 ± 1 °C, relative humidity 75 ± 5%, and a photoperiod of 16 h light: 8 h dark) until they spun cocoons and pupated. The cocoons were then transferred to the moth emergence room until adult emergence. The conditions of the moth emergence room were as follows: temperature 22 ± 1 °C, relative humidity 75 ± 5%, and a photoperiod of 16 h light: 8 h dark.

### 2.5. Assessment of Reproductive Toxicity Phenotypes

#### 2.5.1. Calculation of Gonadosomatic Index in Larvae and Pupae

Samples were collected from the treatment and control groups when the larvae were reared to the 15th day of the fifth instar and the first day after cocooning and pupation. Three biological replicates were performed per group, with three individuals per replicate (three 15-day-old fifth-instar male larvae or three 1-day-old male pupae, respectively). For each replicate, the three individuals were measured individually, and the mean value was used as a single data point per replicate.

The body weight of each individual was measured using a BSA822-CW electronic balance (Sartorius AG, Göttingen, Germany). Subsequently, a pair of testes was dissected out under ice-cold conditions, placed in 1× PBS buffer, and carefully cleaned to remove any adhering tissues. After blotting dry with filter paper, the testes were weighed. The gonadosomatic index (GSI) was calculated according to the following formula [25]: $gonadosomatic\;index\left(GSI\right)(\%)=\frac{gonad\;wet\;weight}{whole\;body\;wet\;weight}\times 100\%$.

#### 2.5.2. Histological Observation of Testicular Tissue in Larvae and Pupae

The testes were collected at the same time points as described in Section 2.5.1. Three biological replicates were performed per group, with three individuals per replicate (three 15-day-old fifth-instar male larvae or three 1-day-old male pupae, respectively). For each replicate, the three individuals were processed separately, and the representative images were selected for morphological analysis.

They were fixed in 4% paraformaldehyde, dehydrated through a graded ethanol series, cleared in xylene, and embedded in paraffin. Sections were cut at a thickness of 4 μm using an RM2016 rotary microtome (Leica Microsystems, Wetzlar, Germany) and stained with hematoxylin and eosin (HE) following standard protocols [26]. Histological observations were performed using an Eclipse Ci microscope (Nikon, Tokyo, Japan), and images were captured for morphological analysis.

#### 2.5.3. Sperm Counting and Morphological Observation in Adult *A. pernyi* Moths

On the second day after the emergence of adult male moths in the treatment and control groups, virgin female moths that had not been exposed to insecticides were selected and mated with them in the mating room, respectively. The conditions of the mating room were as follows: temperature 22 ± 1 °C, relative humidity 75 ± 5%, and constant light during mating. Three biological replicates were performed per group, with three mating pairs per replicate, and all replicates were conducted in mating boxes. For each biological replicate, the three mating pairs were processed individually; sperm counting and morphological observation were performed separately for each pair, and representative images were selected for morphological analysis.

Following a 1 h mating period, the female moths were separated from the males, and the bursa copulatrix of each female was dissected to collect the spermatophore. Subsequently, the spermatophores were punctured to release the sperm, which were diluted 30-fold in Grace’s insect cell culture medium. The diluted sperm suspensions were loaded into a sperm counting chamber, and sperm counting and morphological observations were performed using a ML-500JZ sperm analyzer (Mailang, Tianjin, China) [27].

#### 2.5.4. Determination of Sperm Acrosin Activity in Adult *A. pernyi* Moths

The adult moths were collected as described in Section 2.5.3. Three biological replicates were performed per group, with thirty mating pairs per replicate, and all replicates were conducted in mating baskets. For each biological replicate, the spermatophores collected from all thirty mating pairs were pooled, and an aliquot containing the required number of sperm (as specified by the kit instructions) was taken for acrosin activity measurement.

After mating for 1 h, female moths were separated from the males, and the bursa copulatrix was dissected to collect the spermatophore. Acrosin activity of eupyrene sperm was determined using a sperm acrosin activity assay kit (Ruiaijin Biotechnology, Tianjin, China) (substrate enzyme method) according to the manufacturer’s instructions. Absorbance was measured using a UV2600 UV-visible spectrophotometer (Shimadzu, Kyoto, Japan) [27,28]. Enzyme unit definition and calculation formula: One unit (IU) of acrosin activity was defined as the amount of enzyme required to hydrolyze 1.0 μmol of BAPNA per minute at 24 °C, $Acrosin\;activity\;(\mu IU/10^{6}\;sperm\;count)=\frac{\left(absorbance\;of\;the\;sample\;tube-absorbance\;of\;the\;blank\;tube\right)\times 10^{6}}{247.5\times 10}$.

#### 2.5.5. Assessment of Sperm Motility in Adult *A. pernyi* Moths

The adult moths were collected as described in Section 2.5.3. Three biological replicates were performed per group, with three mating pairs per replicate, and all replicates were conducted in mating boxes. For each replicate, the three mating pairs were processed separately, and the motility parameters were averaged to obtain a single value per replicate.

After mating for 2 h and 50 min, female moths were separated from the males, and the bursa copulatrix was dissected to collect the spermatophore. The spermatophore was punctured to release the sperm, which were then diluted 30-fold with Grace’s insect cell culture medium. After thorough mixing, the diluted sperm suspension was loaded into a sperm counting chamber, and motility parameters of apyrene sperm were analyzed using a ML-500JZ sperm analyzer (Mailang, Tianjin, China) [27,29].

#### 2.5.6. Mating Behavior and Post-Mating Egg Traits

The adult moths were collected as described in Section 2.5.3. Three biological replicates were performed per group, with 30 mating pairs per replicate, and all replicates were conducted in mating baskets. The number of mating pairs was recorded every 10 min for a total observation period of 30 min. Subsequently, after all moth pairs had mated for 12 h, the female moths were separated from the males. From each replicate group, 20 female moths were randomly selected and allowed to oviposit for 24 h. For each biological replicate, the eggs laid by each of the 20 females were assessed individually; the number of eggs laid, hatching rate, dead embryo rate, and unfertilized egg rate were calculated per female, and the mean values of the 20 females were used as a single data point per replicate.

After oviposition, the eggs were incubated under conditions of 23 ± 1 °C, 75 ± 5% relative humidity, and a photoperiod of 16 h light: 8 h dark. Egg traits were assessed 10 days later, including the number of eggs laid, hatching rate, dead embryo rate, and unfertilized egg rate.

### 2.6. Molecular Mechanism of Reproductive Toxicity

#### 2.6.1. RNA Sequencing

First, 15-day-old fifth-instar larvae from both the treatment and control groups were dissected under ice-cold conditions in a laminar flow hood. A pair of testes was collected from each larva, carefully cleaned to remove adhering tissues in 1× PBS buffer, rinsed with nuclease-free water, immediately snap-frozen in liquid nitrogen, and stored at −80 °C after 3 h. Three biological replicates were performed per group, with 10 pairs of testes pooled per replicate.

Total RNA was extracted from the pooled testis samples using QIAzol Lysis Reagent (Qiagen, Hilden, Germany). Library preparation was carried out with the Illumina^®^ Stranded mRNA Prep, Ligation (Illumina, San Diego, CA, USA), followed by sequencing on the DNBSEQ-T7 platform to generate 150 bp paired-end reads, achieving a sequencing depth of at least 7.09 Gb per sample (total 44.97 Gb of clean data).

Raw sequencing data underwent quality control using fastp software version 0.19.5 (https://github.com/OpenGene/fastp, accessed on 6 June 2025) [30]. Reads were mapped to the reference genome GWHABGR00000000 (https://ngdc.cncb.ac.cn/gwh, accessed on 6 June 2025) [31] with HISAT2 software version 2.2.1 (https://daehwankimlab.github.io/hisat2/, accessed on 6 June 2025) [32]. Expression levels were quantified by RSEM version 1.3.3 (http://deweylab.github.io/RSEM, accessed on 6 June 2025/) [33]. Differential expression analysis was performed using DESeq2 software version 1.12.3 (http://bioconductor.org/packages/stats/bioc/DESeq2/, accessed on 6 June 2025) [34], applying thresholds of |log_2_FC| > 1 and a false discovery rate (FDR) < 0.01 to identify differentially expressed genes (DEGs).

Enrichment analysis of the DEGs was conducted based on Gene Ontology (GO) (http://geneontology.org/, accessed on 6 June 2025) [35] and the Kyoto Encyclopedia of Genes and Genomes (KEGG) (https://www.genome.jp/kegg/, accessed on 6 June 2025) [36] databases. The clusterProfiler software version 4.0 was employed for over-representation analysis using a hypergeometric test [37]. The background gene set consisted of all genes expressed in the testis (based on the RNA-seq data). The resulting *p*-values were adjusted for multiple testing using the Benjamini–Hochberg (BH) procedure to control the false discovery rate (FDR). GO terms or KEGG pathways with an adjusted *p*-value (FDR) < 0.05 were considered significantly enriched.

#### 2.6.2. RT-qPCR Validation

Candidate DEGs were selected from the transcriptomic data, and their primers were designed by Primer3 (https://primer3.ut.ee/, accessed on 6 June 2025) (Table S2). For cDNA synthesis, total RNA was reverse transcribed with the TransScript First-Strand cDNA Synthesis kit (AiDLAB Biotech, Beijing, China). RT-qPCR was subsequently carried out with the SYBR Green QPCR Mix (Sichuan KeJin, Deyang, China). Based on the results of our pre-experiment, *β-actin* was chosen as the internal reference gene, and relative expression levels were calculated using the 2^−△△ Ct^ method [38].

### 2.7. Data Analysis

The median lethal concentration (LC_50_) and its 95% confidence limits (95% CL) were determined using PoloPlus 1.0 software [39]. P-values were analyzed with GraphPad InStat 3.0 software. Mean values and standard errors were statistically processed using SPSS 25.0 software. Differences between the treatment and control groups were assessed by an independent samples *t*-test, with significance levels set at *p* < 0.05 (significant) and *p* < 0.01 (highly significant).

## 3. Results

### 3.1. Bioassay of Fifth-Instar Male A. pernyi Larvae

The result of the laboratory toxicity bioassay of β-cypermethrin against fifth-instar male *A. pernyi* larvae is presented in Table 1. The LC_50_ value of β-cypermethrin for the Liaocanda 9 strain was 0.020 mg/L.

### 3.2. Effects of β-Cypermethrin Exposure on the Gonadosomatic Index of Male A. pernyi Larvae and Pupae

**Figure 1:**
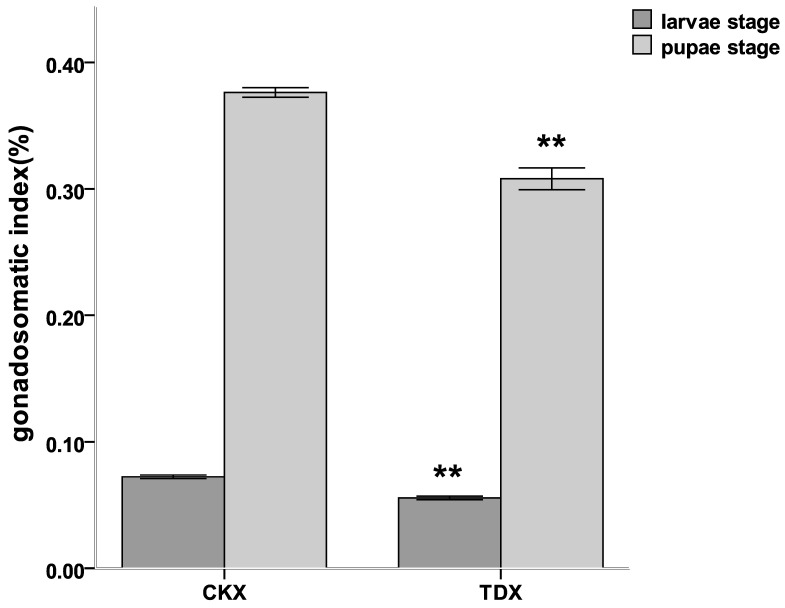
Effects of sublethal β-cypermethrin exposure on gonadosomatic index (GSI) of *A. pernyi*. Dark-colored bars represent 15-day-old fifth-instar male larvae, and light-colored bars represent 1-day-old male pupae. Error bars represent the standard error of the mean (SEM). ** *p* < 0.01 (independent samples *t*-test). CKX, control group; TDX, LC_20_ β-cypermethrin treatment group. *n* = 3 biological replicates.

The gonadosomatic index (GSI) values of 15-day-old fifth-instar male larvae and 1-day-old male pupae are shown in Figure 1. Compared with the control group (CKX), the GSI values of both larvae and pupae in the LC_20_ β-cypermethrin treatment group (TDX) were highly significantly decreased (*p* < 0.01). These results indicated that sublethal exposure to β-cypermethrin delayed testicular development in male larvae and that this retarding effect persisted through metamorphosis into the pupal stage.

### 3.3. Effects of β-Cypermethrin Exposure on Testicular Structure in Male A. pernyi Larvae and Pupae

**Figure 2:**
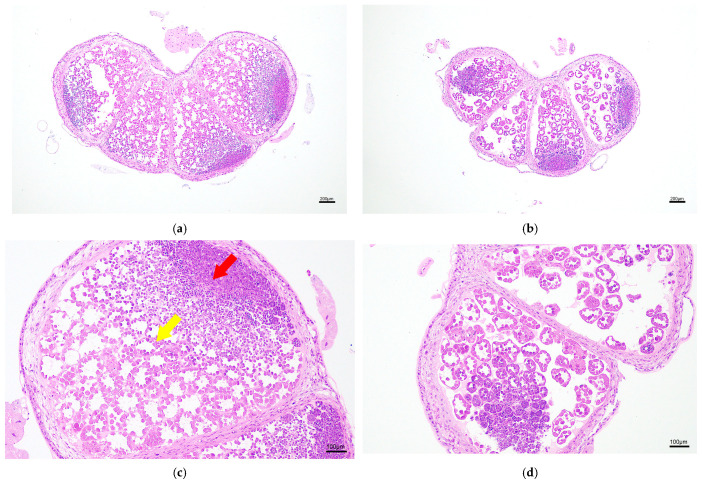

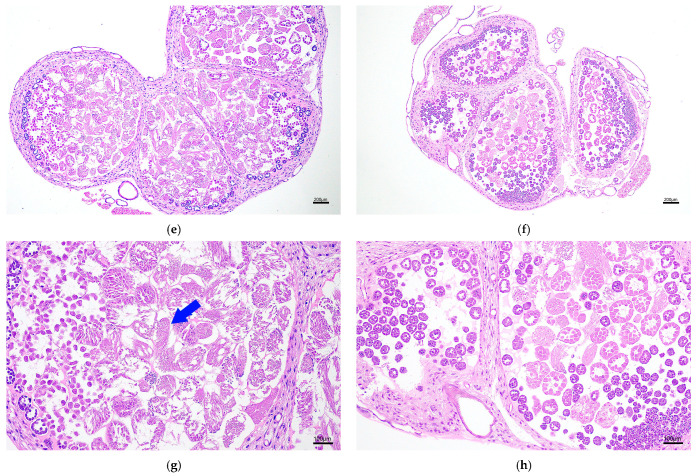
Effects of sublethal β-cypermethrin exposure on testicular histomorphology in *A. pernyi*. (**a**,**c**) Control 15-day-old fifth-instar larvae. (**b**,**d**) LC_20_ β-cypermethrin-treated 15-day-old fifth-instar larvae. (**e**,**g**) Control 1-day-old pupae. (**f**,**h**) LC_20_ β-cypermethrin-treated 1-day-old pupae. The red arrow indicates the spermatogonia region, the yellow arrow indicates the spermatocytes region, and the blue arrow indicates sperm bundles. Scale bars = 200 μm (**a**,**b**,**e**,**f**) and 100 μm (**c**,**d**,**g**,**h**).

The testicular structure of 15-day-old fifth-instar larvae is shown in Figure 2a–d. In the control group (Figure 2a,c), the testis was kidney-shaped, with the inner membrane extending inward to form three septa that divided the testis into four relatively uniform testicular chambers. Within each chamber, spermatogonia and spermatocytes were arranged in an orderly gradient from the apical to the basal region. Germ cells at the same developmental stage were enclosed within the same spermatocyst, and the spermatocysts were arranged in a compact and orderly manner with clearly defined boundaries.

Compared with the control group, the LC_20_ β-cypermethrin treatment group (Figure 2b,d) showed a marked reduction in testicular volume, a decreased number of spermatocysts, a disorganized arrangement of spermatocysts, and significantly enlarged intercystic spaces. These results indicated that sublethal β-cypermethrin exposure not only inhibited overall testicular growth and development but also disrupted the normal progression of germ cell differentiation, leading to the loss or degeneration of some germ cells during development, thereby disturbing their orderly arrangement and ultimately resulting in a looser testicular tissue structure.

The testicular structure of 1-day-old male pupae is shown in Figure 2e–h. In the control group (Figure 2e,g), the pupal testis was morphologically similar to that of the larval stage but significantly enlarged. Spermatids within most spermatocysts had elongated and formed sperm bundles.

Compared with the control group, the LC_20_ β-cypermethrin treatment group (Figure 2f,h) exhibited similar abnormalities as observed in the larval stage, including reduced testicular volume, a decreased number of spermatocysts, disorganized arrangement, and enlarged intercystic spaces. Moreover, testicular deformity was also observed, and spermatocyst development was markedly delayed, with only a few spermatocysts developing into sperm bundles. These results indicated that the inhibitory effects of sublethal β-cypermethrin exposure on testicular development and the disruption of germ cell differentiation persisted into the pupal stage, ultimately impairing the terminal phase of spermatogenesis—spermiogenesis.

### 3.4. Effects of β-Cypermethrin Exposure on Sperm Quantity and Morphology in Adult A. pernyi Moths

Dissection of the bursa copulatrix of female moths revealed that spermatophores in the control group measured approximately 2.5 mm in diameter, whereas those in the treatment group measured only about 2.0 mm, indicating that sublethal β-cypermethrin exposure significantly reduced the ejaculate volume in male moths. After puncturing the spermatophore to release the semen, the sperm were observed and counted using a Mailang sperm analyzer. Approximately 2400 eupyrene sperm bundles were counted per spermatophore in the control group, whereas only about 1800 were counted in the treatment group (approximately 25% reduction).

**Figure 3:**
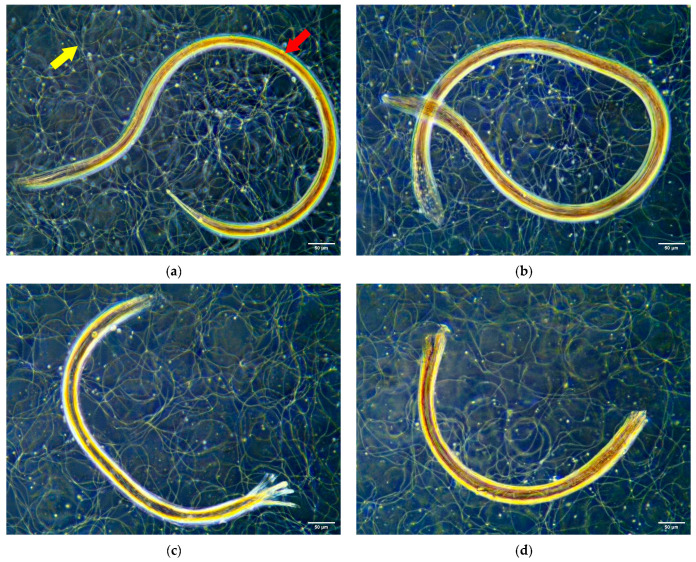
Effects of sublethal β-cypermethrin exposure on sperm morphology in adult *A. pernyi*. (**a**,**b**) Sperm morphology in the control group. (**c**,**d**) Sperm morphology in the LC_20_ β-cypermethrin treatment group. The red arrow indicates eupyrene sperm bundles, and the yellow arrow indicates apyrene sperm. Scale bars = 50 μm.

Sperm morphology is shown in Figure 3. The eupyrene sperm bundles measured approximately 1200–1400 μm in the control group (Figure 3a,b), but were significantly shorter, at approximately 650–750 μm, in the treatment group (Figure 3c,d).

The reduction in the number of eupyrene sperm bundles in the treatment group was highly consistent with the morphological damage observed in testicular tissue sections from the larval and pupal stages, including a decreased number of spermatocysts and impaired spermiogenesis. This indicated that sublethal β-cypermethrin exposure led to substantial loss of germ cells during development, resulting in decreased mature sperm production. Furthermore, the significant shortening of eupyrene sperm bundles provided additional evidence that the exposure induced sperm morphological abnormalities.

### 3.5. Effects of β-Cypermethrin Exposure on Sperm Function in Adult A. pernyi Moths

**Figure 4:**
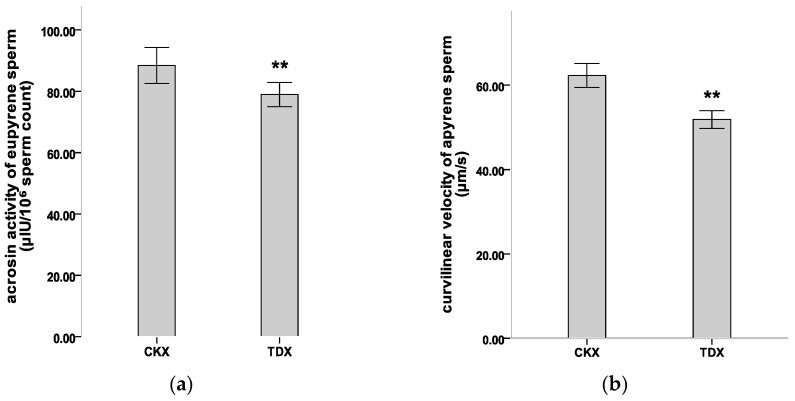
Effects of sublethal β-cypermethrin exposure on sperm functional parameters in *A. pernyi*. (**a**) Acrosin activity of eupyrene sperm. (**b**) Curvilinear velocity (VCL) of apyrene sperm. Error bars represent the standard error of the mean (SEM). ** *p* < 0.01 (independent samples *t*-test). CKX, control group; TDX, LC_20_ β-cypermethrin treatment group. *n* = 3 biological replicates.

*A. pernyi* produces two types of sperm: eupyrene (nucleated) and apyrene (anucleated) sperm. Sperm function is collectively determined by the acrosin activity of eupyrene sperm and the motility of apyrene sperm. Apyrene sperm exhibit a helical wave-like movement pattern, and curvilinear velocity (VCL) is a key parameter reflecting their motility. The results are presented in Figure 4. Compared with the control group, both the acrosin activity of eupyrene sperm (Figure 4a) and the curvilinear velocity of apyrene sperm (Figure 4b) were highly significantly decreased (*p* < 0.01) in the LC_20_ β-cypermethrin treatment group, by 10.72% and 16.75%, respectively. These findings indicate that sublethal β-cypermethrin exposure significantly suppressed key functional parameters of both sperm types, simultaneously compromising the fertilization potential of eupyrene sperm and the motility of apyrene sperm.

### 3.6. Effects of β-Cypermethrin Exposure on Mating Behavior and Post-Mating Egg Traits in Adult A. pernyi Moths

**Figure 5:**
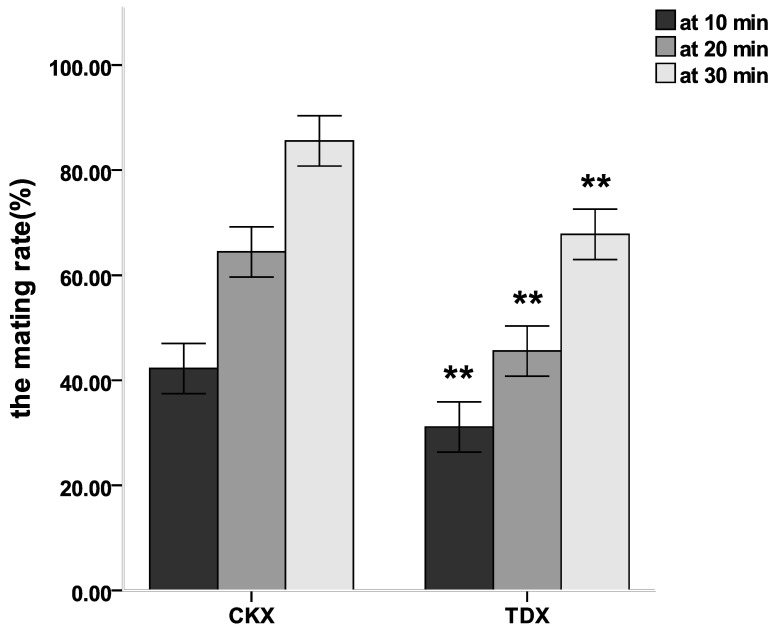
Effects of sublethal β-cypermethrin exposure on mating rate in *A. pernyi.* Dark-colored bars represent the mating rate recorded at 10 min, medium-colored bars at 20 min, and light-colored bars at 30 min. Error bars represent the standard error of the mean (SEM). ** *p* < 0.01 (independent samples *t*-test). CKX, control group; TDX, LC_20_ β-cypermethrin treatment group. *n* = 3 biological replicates.

The mating rate results are shown in Figure 5. During the observation period from 10 to 30 min, the mating rate in the LC_20_ β-cypermethrin treatment group was consistently and significantly lower than that in the control group. Compared with the control group, the mating rate decreased by 11.11 percentage points at 10 min and by 18.88 and 17.78 percentage points at 20 and 30 min, respectively. These results indicate that sublethal β-cypermethrin exposure significantly reduced the mating willingness of male *A. pernyi* moths.

The results of the egg trait survey are presented in Table 2. Compared with the control group, the treatment group showed highly significant differences in the number of eggs laid per female, hatching rate, and unfertilized egg rate (*p* < 0.01). Specifically, the number of eggs laid per female in the treatment group was 20.45% lower than that in the control group, the hatching rate decreased by 3.66 percentage points, and the unfertilized egg rate increased by 3.62 percentage points. No significant difference was observed in the dead embryo rate between the two groups, indicating that the reduction in hatching rate in the treatment group was primarily due to increased unfertilized eggs.

These results demonstrate that sublethal β-cypermethrin exposure highly significantly affects both the reproductive output of mated females and the success of egg fertilization, ultimately leading to a decline in overall population fecundity.

### 3.7. Transcriptome Analysis

Transcriptome sequencing was performed on a total of six samples from the control and treatment groups, generating 44.97 Gb of high-quality clean data. Each sample yielded at least 7.09 Gb of clean data, with Q30 base percentages ≥ 95.58%, and the GC content ranged from 43.34% to 44.44%. The alignment efficiency of clean reads from the six samples to the reference genome ranged from 86.14% to 87.93% (Table S3), indicating that the sequencing data and the selected reference genome were suitable for subsequent analysis.

**Figure 6:**
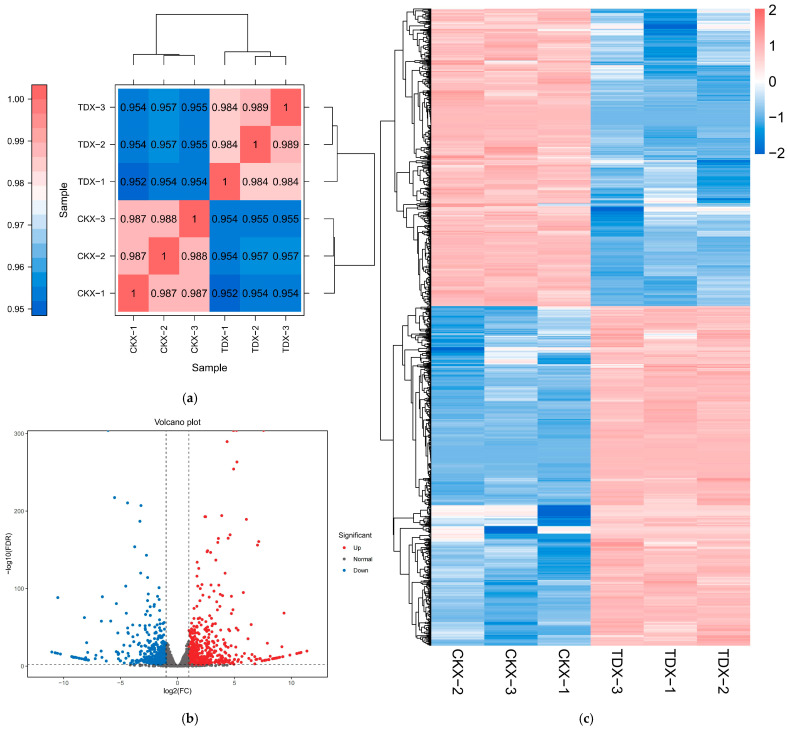
Transcriptome analysis of *A. pernyi* testes in response to β-cypermethrin. (**a**) Correlation clustering analysis plot of samples. (**b**) Volcano map of DEGs distribution (FDR < 0.01, |log_2_FC| > 1); red: up-regulated, blue: down-regulated, gray: not significant. (**c**) Clustering heatmap of DEGs; red: high expression, blue: low expression. *n* = 3 biological replicates.

Pearson correlation analysis of gene expression levels between samples (Figure 6a) revealed high consistency among biological replicates within each group (Pearson’s *r* ≥ 0.984), while intergroup correlations (Pearson’s *r* ≥ 0.952) were lower than intragroup correlations, indicating that LC_20_ β-cypermethrin treatment induced changes in gene expression in *A. pernyi* testis, resulting in a distinct transcriptomic profile compared with the control group.

Differentially expressed genes (DEGs) between the treatment and control groups were identified using thresholds of |log_2_FC| > 1 and FDR < 0.01. A total of 1193 DEGs were identified, including 636 up-regulated and 557 down-regulated genes (Figure 6b). Based on these DEGs, the three replicate samples in each group exhibited a clear clustering pattern (Figure 6c).

### 3.8. GO and KEGG Enrichment Analysis of DEGs

**Figure 7:**
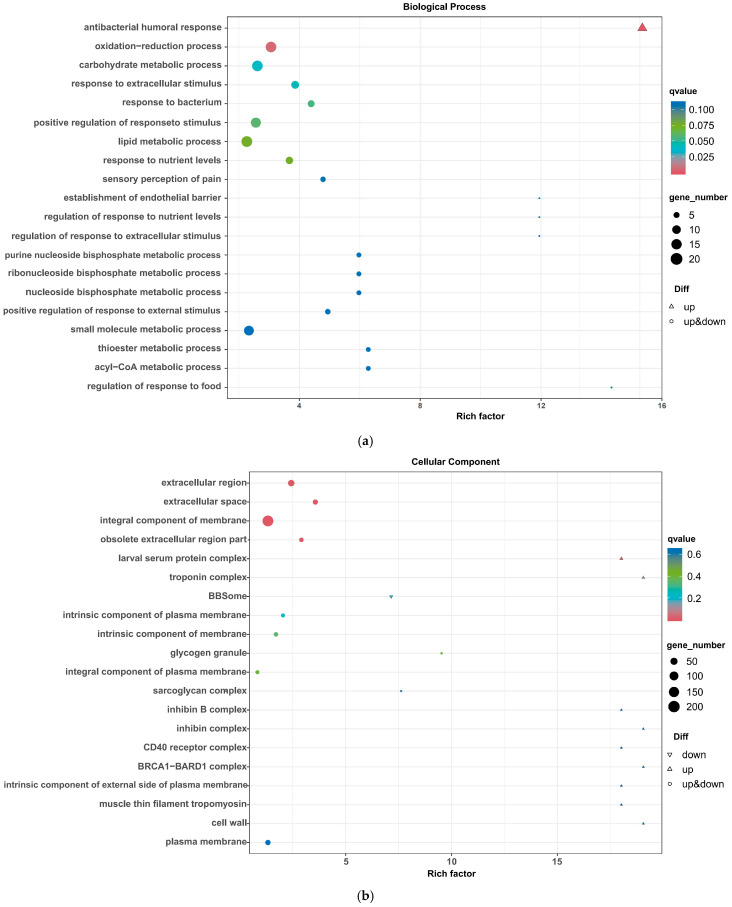

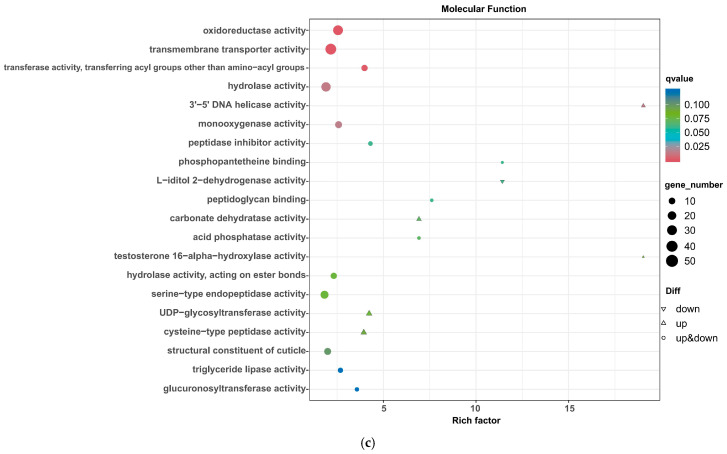
GO functional enrichment analysis of DEGs. (**a**) Biological process (BP). (**b**) Cellular component (CC). (**c**) Molecular function (MF). The significance of GO terms is indicated by the q-value (color bar) and the rich factor (*x*-axis), while the circle size represents the number of DEGs.

To elucidate the functional categories of differentially expressed genes (DEGs) in the testis under LC_20_ β-cypermethrin exposure, GO enrichment analysis was performed (Figure 7). In the Biological Process category (Figure 7a), DEGs were significantly enriched in terms such as “oxidation–reduction process” (GO:0055114) and “carbohydrate metabolic process” (GO:0005975). Cellular component analysis (Figure 7b) revealed significant enrichment in “extracellular region” (GO:0005576) and “integral component of membrane” (GO:0016021). In the molecular function category (Figure 7c), significantly enriched terms mainly included “oxidoreductase activity” (GO:0016491), “transmembrane transporter activity” (GO:0022857), “3′–5′ DNA helicase activity” (GO:0043138), “hydrolase activity” (GO:0016787), and “monooxygenase activity” (GO:0004497).

The enrichment of these functional categories suggests that LC_20_ β-cypermethrin exposure may disrupt redox homeostasis and energy metabolism in the testis, as well as affect membrane structure and transmembrane transport functions. These molecular changes are closely associated with testicular development and spermatogenesis. Notably, the significant enrichment of DNA helicase activity provides a clue that genome integrity may be compromised, offering insights for further exploration of the underlying mechanisms of reproductive toxicity.

**Figure 8:**
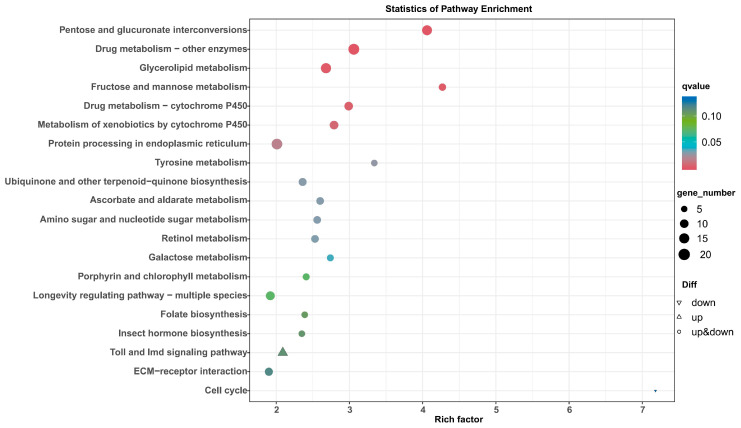
KEGG pathway enrichment analysis of DEGs. The significance of pathways is indicated by the q-value (color bar) and the rich factor (*x*-axis), while the circle size represents the number of DEGs.

To investigate the metabolic pathways involved in the response to LC_20_ β-cypermethrin exposure in the testis, KEGG enrichment analysis was performed on the differentially expressed genes (DEGs). The 20 most significantly enriched pathways are shown in Figure 8, and these pathways can be categorized into the following functional groups:

Xenobiotic degradation and detoxification-related pathways: These included “Drug metabolism–other enzymes” (ko00983), “Drug metabolism–cytochrome P450” (ko00982), and “Metabolism of xenobiotics by cytochrome P450” (ko00980). Enrichment of these pathways suggests that the testis responded to pesticide stress by activating detoxification pathways.

Carbohydrate and energy metabolism-related pathways: Several sugar metabolism pathways were significantly enriched, including “Pentose and glucuronate interconversions” (ko00040), “Fructose and mannose metabolism” (ko00051), and “Glycerolipid metabolism” (ko00561). These changes indicate disruption of basal energy metabolism, which may affect energy supply during spermatogenesis.

Protein processing and stress response pathways: “Protein processing in endoplasmic reticulum” (ko04141) was significantly enriched, suggesting that unfolded protein responses or endoplasmic reticulum homeostasis may be perturbed, which is associated with protein quality control and cellular stress.

In summary, KEGG enrichment analysis revealed that LC_20_ β-cypermethrin exposure primarily affects pathways related to detoxification metabolism, energy balance, and protein homeostasis in the testis. These molecular perturbations likely contribute to the testicular dysfunction observed following exposure.

### 3.9. RT-qPCR Validation of DEGs Related to Reproductive Toxicity

**Figure 9:**
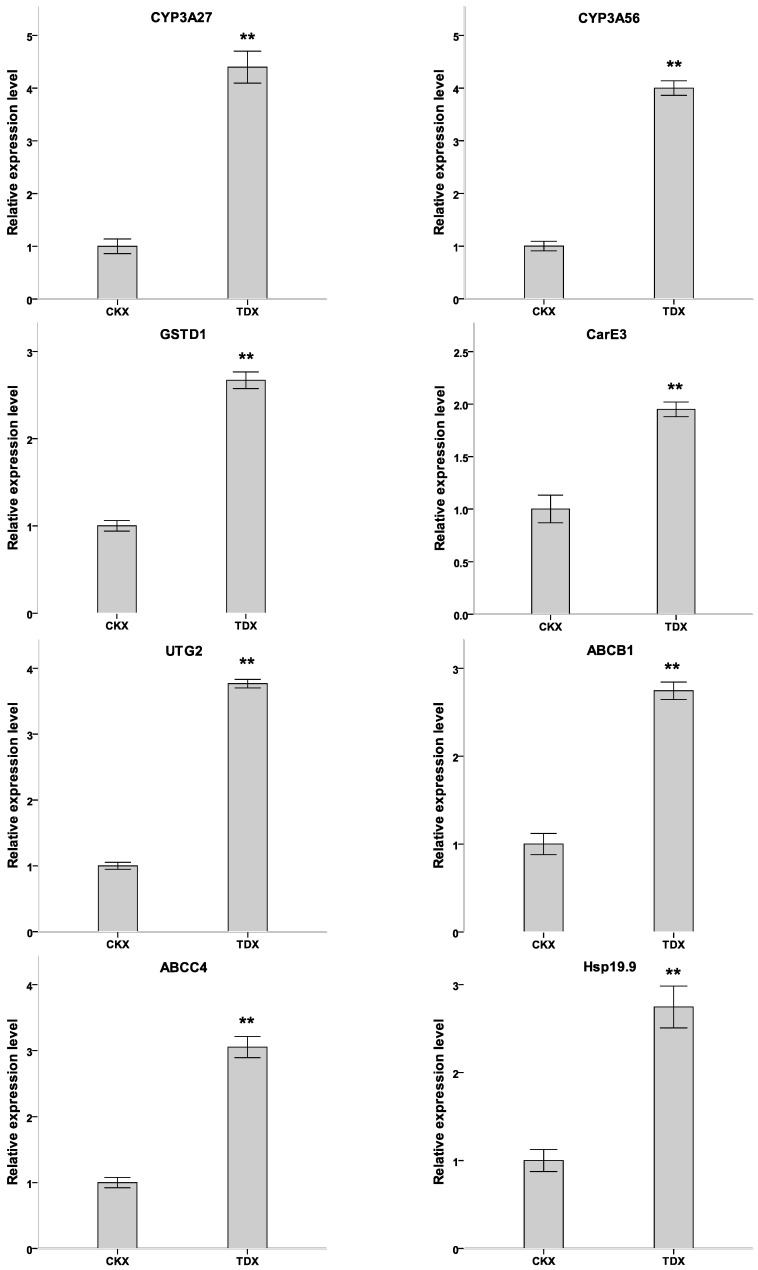

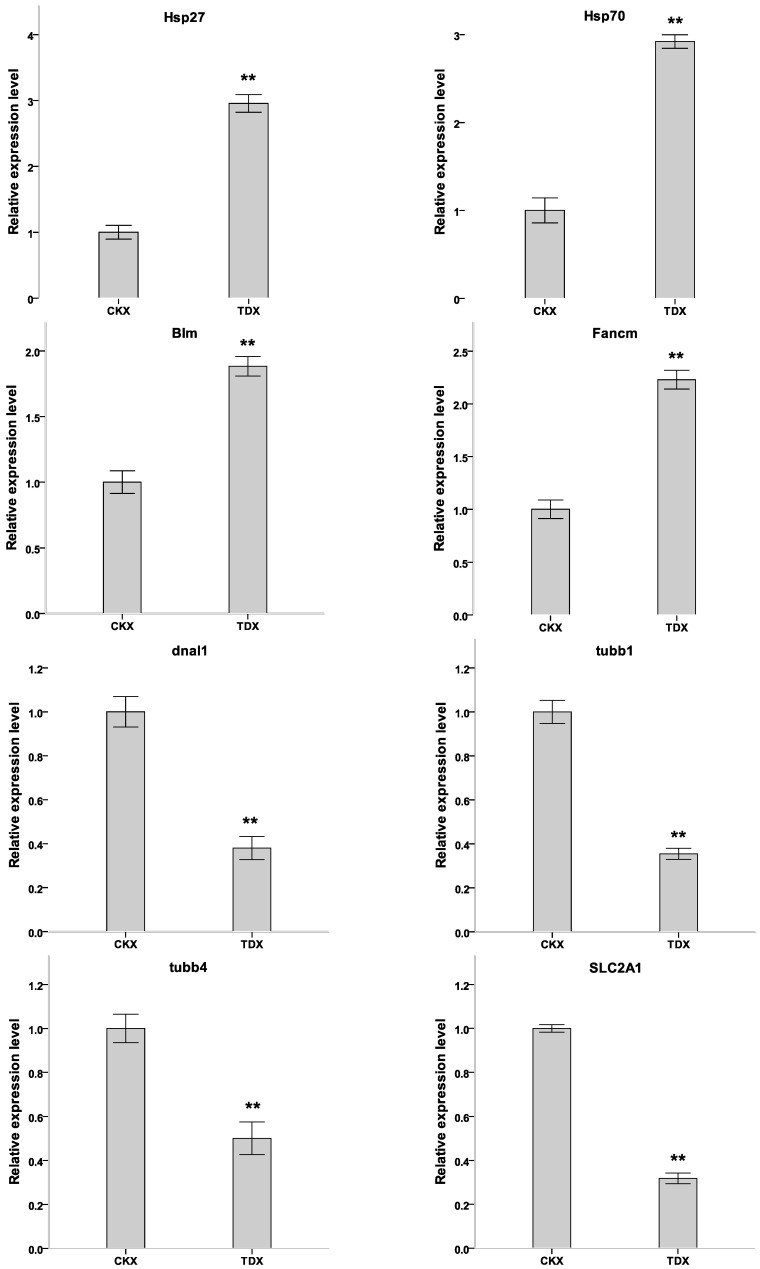

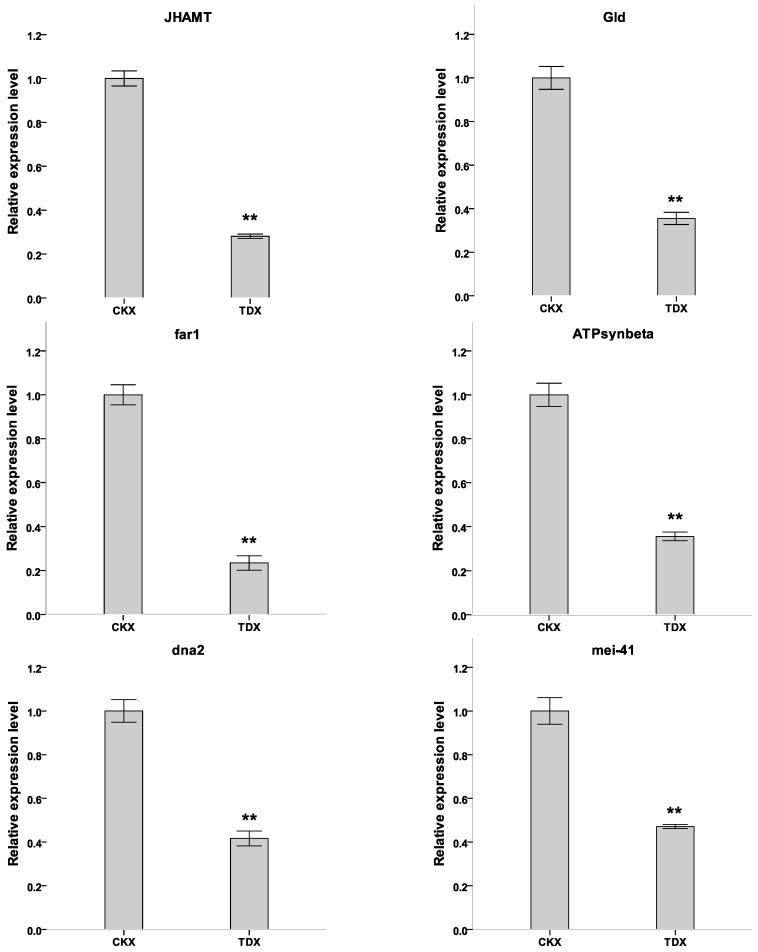
RT-qPCR validation of differentially expressed genes in the testis of *A. pernyi* after β-cypermethrin treatment. Relative expression levels of 22 selected genes in the control (CKX) and LC_20_ β-cypermethrin treatment (TDX) groups. Error bars represent the standard error of the mean (SEM). ** *p* < 0.01 (independent samples *t*-test). The housekeeping gene β-actin was used as an internal control. *n* = 3 biological replicates.

To elucidate the mechanism of male reproductive toxicity induced by sublethal β-cypermethrin exposure in *A. pernyi*, RT-qPCR was used to validate the differentially expressed genes (DEGs) identified from transcriptome sequencing data. These included the up-regulated genes *CYP3A27*, *CYP3A56*, *GSTD1*, *CarE3*, *UGT2*, *ABCB1*, *ABCC4*, *Hsp19.9*, *Hsp27*, *Hsp70*, *Blm*, and *Fancm* and the down-regulated genes *dnal1*, *tubb1*, *tubb4*, *ATPsynbeta*, *SLC2A1*, *far1*, *JHAMT*, *Gld*, *mei-41*, and *dna2* (Figure 9). Compared with the control group, the relative expression levels of these DEGs in the testis of the LC_20_ β-cypermethrin treatment group were up-regulated by 1.88–4.40-fold (*p* < 0.01) and down-regulated to 0.50–0.15-fold (i.e., 50% to 15% of the control levels) (*p* < 0.01). These differential expression patterns reveal the molecular response of the *A. pernyi* testis to β-cypermethrin exposure.

## 4. Discussion

In this study, we systematically evaluated the male reproductive toxicity induced by sublethal exposure to β-cypermethrin in *A. pernyi* and investigated the underlying molecular mechanisms through transcriptome analysis. Our results demonstrated that exposure to the LC_20_ concentration of β-cypermethrin not only significantly suppressed gonadal development, spermatogenesis, and sperm function in the treated males but also led to aberrant mating behavior and further reduced egg production and hatching rates in mated females, ultimately resulting in a decline in overall population fecundity.

Transcriptome analysis revealed an imbalance between “defense activation” and “reproductive inhibition” in the testis, with widespread up-regulation of detoxification and stress response genes and extensive down-regulation of genes critical for reproductive functions. The following sections discuss the key phenotypic and molecular findings in detail.

### 4.1. Reproductive Toxicity Phenotypes Induced by Sublethal β-Cypermethrin Exposure

In this study, sublethal β-cypermethrin exposure caused multi-level, cross-stage damage to male reproduction in *A. pernyi*. Beginning in the larval stage, the treatment group exhibited reduced testicular volume, a decreased number of spermatocysts with disorganized arrangement, and impaired germ cell differentiation. These damages persisted into the pupal stage, ultimately leading to impaired spermatogenesis. In the adult stage, the number of eupyrene sperm bundles was reduced by approximately 25%, and the remaining bundles showed abnormal morphology, becoming shorter in length.

These findings are consistent with previous reports of insecticide-induced testicular volume reduction, structural damage, spermatogenic dysfunction, and significant decreases in the numbers of both eupyrene and apyrene sperm in various insect species, including *Bombyx mori* (Linnaeus, 1758) (Bombycidae) exposed to chlorfenapyr [40], *Spodoptera mauritia* (Boisduval, 1833) (Noctuidae) exposed to flufenoxuron [41], *Spodoptera eridania* (Stoll, 1782) (Noctuidae) exposed to azadirachtin [42], and *Spodoptera litura* (Fabricius, 1775) (Noctuidae) exposed to chlorfluazuron [43].

Regarding sperm function, both the acrosin activity of eupyrene sperm and the curvilinear velocity of apyrene sperm were highly significantly decreased. Behavioral observations revealed that the mating rate of treated male moths was significantly reduced. Furthermore, females mated with treated males exhibited a significant decrease in egg production and hatching rate, along with a significant increase in the unfertilized egg rate. These results indicate that sublethal insecticide stress induces damage across the entire chain from germ cell development to post-mating reproductive success, with persistent and cumulative effects.

### 4.2. Persistent Activation of the Detoxification System and the “Metabolic Exhaustion” State

Transcriptome analysis revealed that multiple xenobiotic metabolism-related genes were significantly up-regulated in the testis of the treatment group. These included phase I metabolic enzymes *CYP3A27* and *CYP3A56*, phase II metabolic enzymes *GSTD1*, *CarE3*, and *UGT2*, as well as phase III efflux transporters *ABCB1* and *ABCC4*. Concurrently, heat shock protein genes, which are key molecules in stress responses, including *Hsp19.9*, *Hsp27*, and *Hsp70*, were also broadly up-regulated. KEGG enrichment analysis further confirmed that multiple detoxification and stress response pathways were significantly enriched. These results indicate that even 13 days after the cessation of stress, the testis maintained a high level of detoxification defense.

Xenobiotic detoxification is a highly energy-demanding process: the cytochrome P450 system requires NADPH as an electron donor, and ABC transporters directly hydrolyze ATP to export metabolites out of cells [44,45]. Similarly, sustained activation of heat shock proteins also consumes substantial amounts of ATP [46]. In parallel, we observed significant enrichment of multiple energy metabolism pathways, along with the widespread down-regulation of energy metabolism genes closely associated with spermatogenesis, such as *ATPsynbeta* and *SLC2A1*.

Based on these findings, we propose the “metabolic exhaustion” hypothesis: following sublethal β-cypermethrin stress, the testis maintains a sustained high-activity state of detoxification and stress response systems, which continuously consumes energy and reducing equivalents. This may deplete resources required for critical processes such as germ cell proliferation, differentiation, and spermiogenesis, leading to insufficient energy supply for spermatogenesis and ultimately resulting in impaired germ cell development and functional defects.

This energetic trade-off is directly reflected in our phenotypic observations. The 16.75% decrease in the curvilinear velocity of apyrene sperm (Figure 4b) is a classic consequence of ATP shortage, as sperm motility is an ATP-consuming process [47]. The 17.78-percentage-point reduction in mating rate is also consistent with an energy deficit, since courtship and copulation are among the most energy-consuming behaviors in insects [48]. Moreover, the 25% reduction in eupyrene sperm bundles and the 10.72% decrease in acrosin activity (Figure 4a) further illustrate the reproductive cost of sustained detoxification activation. Thus, the “detoxification activation–energy depletion–reproductive suppression” axis not only explains the molecular changes but also accounts for the observed multi-level reproductive impairments.

Previous studies have shown that the allocation trade-off between detoxification metabolism and reproduction is a common strategy for insects to cope with environmental stress [49,50,51]. These findings collectively support the “detoxification activation–energy depletion–reproductive suppression” molecular mechanism model proposed in this study.

It is noteworthy that several core detoxification genes are also involved in endogenous hormone regulation. For example, GO annotations indicate that *CYP3A27* and *CYP3A56* possess “testosterone hydroxylase activity” and are involved in “steroid metabolic process” and “androgen metabolic process”, suggesting that while enhancing detoxification capacity, they may non-specifically interfere with the metabolic balance of androgen-like hormones in *A. pernyi*. *ABCB1* functions not only as an efflux pump but also participates in “hormone transport”.

Previous studies have highlighted that while insect CYP450 family genes play a critical role in insecticide detoxification, their interference with hormone homeostasis may constitute a nontarget effect [52]. Recent functional evidence directly supports this role. Kim et al. [53] showed that *CYP337B5* in *Spodoptera frugiperda* (J.E. Smith, 1797) (Noctuidae) efficiently metabolizes an insecticide while simultaneously participating in juvenile hormone biosynthesis.

Furthermore, the conserved role of P450s in regulating reproductive hormones is underscored by the finding that Li et al. [54] demonstrated that silencing *CYP12A2* in *Mythimna separata* (Walker, 1865) (Noctuidae) reduces 20-hydroxyecdysone titers and impairs reproduction. Therefore, activation of detoxification genes may act as a “double-edged sword”: while providing protection, it may indirectly induce reproductive toxicity through metabolic interference.

### 4.3. Energy Metabolism Disruption and Impaired Spermatogenesis

Spermatogenesis is a highly energy-intensive process in which meiosis, sperm flagellum assembly, and the acquisition of motility all depend on an adequate supply of ATP [55]. In this study, *ATPsynbeta*, a core component of mitochondrial oxidative phosphorylation, was significantly down-regulated. Studies in *D. melanogaster* have provided direct functional evidence for this observation. Yu et al. [55] demonstrated that knockdown of this gene in germ cells led to germ cell maturation arrest and complete male infertility, while somatic hub and cyst cells remained unaffected, underscoring a cell-autonomous requirement for *ATP synthase* in the germline.

Furthermore, Chen et al. [56] showed that knockdown of this gene in the testis caused abnormal spermatogenesis, disrupted nuclear bundles, and male infertility, consistent with a role in late-stage germ cell differentiation. In addition, we observed a significant down-regulation of *SLC2A1*. Under normal physiological conditions, the down-regulation of *SLC2A1* is a hallmark of orderly meiotic entry in germ cells [57].

However, in the present study, the pronounced down-regulation of *SLC2A1* occurred in the testis after pesticide exposure and was accompanied by phenotypes such as impaired spermatogenesis and disrupted germ cell differentiation. This suggests that β-cypermethrin stress may interfere with the normal meiotic initiation program in the testis, leading to premature or excessive down-regulation of *SLC2A1*, thereby affecting glucose uptake capacity, causing insufficient energy supply, and ultimately hindering the progression of spermatogenesis.

In *Locusta migratoria* (Linnaeus, 1758) (Acrididae), Wang et al. [58] demonstrated that knockdown of the SLC2A1 homolog Glut4 similarly led to reduced energy reserves and increased sensitivity to deltamethrin-induced energy depletion. The systemic down-regulation of these energy metabolism genes is highly consistent with the observed phenotypes, including a reduction in the number of eupyrene sperm bundles, uneven sperm length, and decreased motility of apyrene sperm.

### 4.4. Cytoskeletal Damage and Abnormal Sperm Morphology

In this study, cytoskeleton-related genes, including *dnal1*, *tubb1* and *tubb4*, were significantly down-regulated. In *D. melanogaster*, Caggese et al. [59] demonstrated that mutations in the dynein light chain gene lead to displacement of the nuclear cap and defective attachment of the flagellar basal body to the nucleus during spermatid differentiation, ultimately resulting in complete male infertility. Li et al. [60] further showed that this gene is also involved in membrane skeleton assembly during sperm elongation, and its mutation can cause sperm tail curling.

Tubulin is a core structural component of the sperm flagellar axoneme, and reduced expression of tubulin directly affects flagellar assembly and motility [61]. Heu et al. [62] demonstrated that in *Lygus hesperus* (Knight, 1917) (Miridae), knockdown or knockout of the testis-specific *Lhβtub2* leads to male sterility, characterized by reduced sperm count and shortened sperm length. Similarly, Sun et al. [63] showed that in *S*. *frugiperda*, knockout of B2t causes complete male infertility by disrupting eupyrene sperm development and migration. Moreover, a recent study in mice demonstrated that *TUBB4B* is essential for the expansion of differentiating spermatogonia; its loss leads to impaired spermatogonial proliferation and complete male infertility [64].

Therefore, the down-regulation of *tubb1*/*tubb4* observed in this study may disrupt normal spermatogonial proliferation and flagellar assembly, leading to a reduced number of eupyrene sperm bundles and morphological abnormalities. Since the motility of apyrene sperm also depends on the integrity of the flagellar axoneme, the aberrant expression of these cytoskeletal genes inevitably affects apyrene sperm motility. These molecular changes directly account for the shortened length of sperm bundles, as well as the significantly decreased curvilinear velocity of apyrene sperm observed in the treatment group.

### 4.5. Lipid Metabolism Disruption and Damage to Germ Cell Membrane Structure

In this study, *far1* was significantly down-regulated. *far1* encodes a key rate-limiting enzyme in the ether lipid biosynthesis pathway. Ether lipids are essential structural components of sperm cell membranes and cytoplasmic bridges, playing a critical role in maintaining synchronous germ cell development and membrane integrity [65]. *FAR1* is also responsible for the synthesis of seminolipids—a testis-specific subclass of ether lipids. These specialized fats are found exclusively in developing sperm and are essential for their formation and function [66]. In mice, Pan et al. [65] and Tamazawa et al. [66] demonstrated that *FAR1* deficiency leads to reduced testis size, arrest of spermatogenesis, and complete male infertility.

The essential role of ether lipids in reproduction is evolutionarily conserved. In *Caenorhabditis elegans* (Maupas, 1900) (Rhabditidae), Shi et al. [67] showed that loss of *fard-1*, the ortholog of mammalian *FAR1*, results in complete ether lipid deficiency and significantly reduced fertility, along with shortened lifespan and increased sensitivity to oxidative stress. In *Nilaparvata lugens* (Stål, 1854) (Delphacidae), Li et al. [68] reported that RNAi-mediated knockdown of eight *NlFAR* genes, including *NlFAR1*, resulted in female adult infertility, and several *NlFAR* genes were highly expressed in the testis, suggesting a conserved role of FAR family genes in insect reproduction.

Therefore, the down-regulation of *far1* observed in this study may interfere with ether lipid and seminolipid synthesis, thereby destabilizing the cytoplasmic bridges between germ cells, compromising sperm membrane integrity, and impeding the synchronous progression of spermatogenesis. This mechanism is highly consistent with the morphological abnormalities observed in testicular tissue sections from the treatment group, including the disorganized arrangement of spermatocysts and enlarged intercystic spaces, as well as the reduced number of eupyrene sperm bundles.

### 4.6. Meiotic DNA Damage Response and Repair Defects and Fertilization Failure

During meiosis, germ cells generate programmed DNA double-strand breaks to initiate homologous recombination, a process that heavily depends on a sophisticated DNA damage response and repair network [69,70]. In this study, both *mei-41* and *dna2* were significantly down-regulated. Hari et al. [71] identified *mei-41* as the *D. melanogaster* ortholog of ATR kinase, a core kinase in the meiotic DNA damage response, responsible for monitoring DNA double-strand breaks and recruiting repair proteins; it is essential for proper homologous chromosome pairing and DNA break repair. Sekelsky [72] further described *mei-41* as a pleiotropic locus affecting DNA repair, chromosome stability, and replication control, and LaRocque et al. [73] demonstrated that loss of *mei-41* leads to defective homologous recombination repair and impaired cell-cycle regulation.

*dna2* possesses both helicase and nuclease activities and is involved in end resection of DNA double-strand breaks, replication fork maintenance, and telomere maintenance [74]. In *C*. *elegans*, Lee et al. [75] showed that loss of dna-2 function causes aberrant germ cell division rates and defects in germline development. The simultaneous down-regulation of *mei-41* and *dna2* severely impairs the sensing and execution of meiotic DNA damage repair.

Concurrently, GO enrichment analysis revealed significant enrichment of the term “3′–5′ DNA helicase activity”, and the involved genes *Blm* and *Fancm* were both markedly up-regulated, suggesting that testicular cells attempt to partially compensate for the compromised repair function by up-regulating these two genes. However, this compensatory mechanism has a fundamental limitation: *Blm* and *Fancm* possess only helicase activity and lack the kinase signaling function of *mei-41* and the nuclease function of *dna2* and thus cannot overcome the core repair deficiency.

Consequently, the accumulated DNA double-strand breaks during meiosis cannot be properly repaired, leading to chromosome nondisjunction and the massive production of aneuploid sperm. The highly significant increase in the unfertilized egg rate observed in this study may be partially attributable to fertilization failure resulting from accumulated DNA damage and the production of aneuploid sperm.

### 4.7. Hormonal Signaling Disruption and Aberrant Mating Behavior

Juvenile hormone (JH) plays a critical regulatory role in insect reproductive maturation and mating behavior [76]. In this study, the gene encoding the JH synthesis rate-limiting enzyme, *JHAMT*, was significantly down-regulated. In *D. melanogaster*, Wijesekera et al. [77] demonstrated that knockdown of *JHAMT* leads to a marked reduction in male courtship behavior, a defect that can be fully rescued by treatment with a juvenile hormone analog.

In *Tribolium castaneum* (Herbst, 1797) (Tenebrionidae), Parthasarathy et al. [78] showed that RNAi-mediated knockdown of *JHAMT* reduced accessory gland size and Acp gene expression, leading to reduced mating vigor, poor sperm transfer, and decreased female fecundity. Consistent with these findings, we observed a highly significant decrease in mating rate in treated male moths, accompanied by a highly significant reduction in egg production in mated females.

Ecdysone is a key hormone regulating insect metamorphosis and reproductive maturation, and its metabolic homeostasis is essential for spermatogenesis and testicular development [79]. In this study, *Gld* was significantly down-regulated. In insects, *Gld* was newly identified as an ecdysone oxidase capable of metabolizing ecdysone and 20-hydroxyecdysone by Guo et al. [80].

Dong et al. [81] further confirmed that *Gld* functions as a 20E-degrading enzyme, and its down-regulation leads to increased 20E titers. Moreover, in *B*. *mori*, Yamamoto and Nagaoka [82] identified a *Gld* homolog (*bmEO2*) that is abundantly expressed in the larval testis, suggesting a local role in ecdysone metabolism during spermatogenesis. Therefore, the down-regulation of *Gld* may disrupt local ecdysone metabolism in the testis, thereby contributing to the impairment of spermatogenesis observed in treated males.

### 4.8. Integrated Mechanistic Model and Ecotoxicological Implications

Integrating the above analyses, we propose a “dual-hit” mechanism underlying the male reproductive toxicity induced by sublethal β-cypermethrin exposure in *A. pernyi*. Pesticide stress drives the testis into an imbalanced state characterized by “defense activation” and “reproductive inhibition”. On one hand, detoxification-related genes (*CYP3A27*, *CYP3A56*, *CarE3*, *GSTD1*, *UGT2*, *ABCB1*, and *ABCC4*) and heat shock proteins (*Hsp19.9*, *Hsp27*, and *Hsp70*) are significantly up-regulated, indicating that the testis actively clears residual toxicants and responds to proteotoxic stress. This up-regulation likely contributes to enhanced detoxification capacity and stress tolerance in the testis, mitigating tissue damage under sublethal exposure. However, this sustained defensive activation is energetically costly and contributes to a state of “metabolic exhaustion”, which further compromises reproductive functions.

On the other hand, genes critical for reproductive functions are broadly suppressed, including those involved in cytoskeletal structure (*dnal1*, *tubb1*, and *tubb4*), energy metabolism (*ATPsynbeta* and *SLC2A1*), lipid metabolism (*far1*), hormonal regulation (*JHAMT* and *Gld*), and DNA damage response and repair (*mei-41* and *dna2*). This molecular impairment persists even after a 13-day recovery period, suggesting that pesticide exposure during the critical developmental window of the fifth instar causes lasting damage to the testis.

It is important to acknowledge that the transcriptomic and qRT-PCR findings presented here are largely correlative. While the strong association between gene expression changes and reproductive phenotypes supports our proposed mechanism, it does not directly prove causation. Therefore, the inferred roles of the identified pathways and genes require further functional validation. Future studies should include direct measurements of oxidative stress markers (e.g., reactive oxygen species, malondialdehyde, and antioxidant enzyme activities) and hormone titers (e.g., juvenile hormone and ecdysone), as well as loss-of-function or gain-of-function analyses of candidate genes (e.g., *JHAMT*, *ATPsynbeta*, and *far1*) to establish causal links between the molecular alterations and the observed reproductive impairments.

It is noteworthy that several key differentially expressed genes identified in this study, including *dnal1*, *tubb4*, *far1*, and *dna2*, were newly identified and annotated in *A. pernyi* for the first time. To the best of our knowledge, this study represents the first report of the expression changes and potential functions of these genes in pesticide-induced male reproductive toxicity in the Chinese oak silkworm. These findings not only enrich the genomic annotation resources of *A. pernyi* but also provide novel molecular targets for understanding the reproductive toxicology of lepidopteran insects.

In summary, sublethal β-cypermethrin exposure induces male reproductive toxicity in *A. pernyi* through a “dual-hit” mechanism: persistent detoxification activation (causing energy depletion) and direct suppression of reproductive genes collectively impair testicular development, spermatogenesis, sperm function, mating behavior, and offspring reproductive success.

This study systematically elucidates, at the molecular level, the complex mechanisms by which sublethal pesticide exposure induces male reproductive toxicity across developmental stages in insects. These findings provide new scientific evidence for ecological risk assessment of pyrethroid insecticides and offer important insights for the safe use of pesticides in integrated pest management and for developing varieties with superior tolerance traits. Future studies employing RNA interference to validate key genes, together with biochemical assays of oxidative stress and hormone levels, will further clarify the causal relationships underlying β-cypermethrin-induced reproductive toxicity.

## 5. Conclusions

Sublethal β-cypermethrin exposure at the fifth larval instar induced significant and lasting male reproductive toxicity in *A. pernyi*, with damage persisting through the larval, pupal, and adult stages and ultimately reducing reproductive success. Transcriptome analysis revealed that the pathological response involves a “dual-hit” mechanism, i.e., persistent detoxification activation and suppression of reproductive genes.

Nevertheless, this study has certain limitations, including its reliance on correlation-based inferences rather than direct functional validation. Despite this, the present work elucidates the molecular mechanisms underlying sublethal β-cypermethrin-induced male reproductive toxicity in *A. pernyi*. These findings provide a theoretical basis for future research on the reproductive toxicology of economically important lepidopteran insects and for the ecological risk assessment of pyrethroid insecticides. Future studies using RNA interference to validate key genes will further clarify their direct roles in β-cypermethrin-induced reproductive toxicity.

## Figures and Tables

**Table 1:** Toxicity bioassay of β-cypermethrin against fifth-instar male *A. pernyi* larvae.

Insecticides	Strain	Slope ± SE	LC_50_ (95% CL) (mg/L)	χ^2^ (df)	*p*
β-cypermethrin	Liaocanda 9	1.94 ± 0.22	0.020 (0.016–0.024)	9.71 (13)	0.72

**Table 2:** Effects of sublethal β-cypermethrin exposure on egg traits in *A. pernyi*.

	Eggs Laid per Female Moth	Hatching Rate (%)	Unfertilized Egg Rate (%)	Embryo Mortality Rate (%)
CKX	313 ± 2.65	99.52 ± 0.25	0.39 ± 0.15	0.09 ± 0.02
TDX	249 ± 1.53 **	95.86 ± 0.40 **	4.01 ± 0.40 **	0.13 ± 0.01

Note: Data are presented as mean ± SEM. ** *p* < 0.01 (independent samples *t*-test). CKX, control group; TDX, LC_20_ β-cypermethrin treatment group. *n* = 3 biological replicates.

## Data Availability

The data has been submitted to the NCBI database with the accession ID PRJNA1472637. The original contributions presented in this study are included in the article/Supplementary Materials. Further inquiries can be directed toward the corresponding author.

## Supplementary Materials

The following supporting information can be downloaded at https://www.mdpi.com/article/10.3390/insects17060633/s1, Table S1. Sublethal concentration (LC_20_) and probit statistics of β-cypermethrin against fifth-instar male *A. pernyi* larvae; Table S2. RT-qPCR primer information of differentially expressed genes; Table S3. Transcriptome data statistic.
