# Supplementary material for: Mechanisms Underlying Male Reproductive Toxicity Induced by Sublethal β-Cypermethrin Exposure in Antheraea pernyi (Guérin-Méneville, 1855) (Saturniidae)
Source: Insects. 2026 Jun 15;17(6):633. doi: 10.3390/insects17060633 (PMC13299614; doi:10.3390/insects17060633)
Supplement: Supplementary file 1 [file insects-17-00633-s001.zip › insects-4340386-supplementary.pdf]

Mechanisms underlying male reproductive toxicity induced by sublethal beta-cypermethrin exposure in *Antheraea pernyi*

Xin Chen[1], Tianyi Zhang[1], Liang Xu[1], Junshan Chen[1], Peifeng Liu[1], Fengquan Liu[1], MiaoMiao Chen[1], Shiwen Zhao[1], Xisheng Li[1,*]

[1]Sericultural Research Institute of Liaoning Province, Dandong 118100, China

[*]Corresponding Author: Xisheng Li

Table S1. Sublethal concentration (LC_{20}) and Probit statistics of β-cypermethrin against fifth-instar male *A. pernyi* larvae

Insecticides	Strain	Slope ± SE	LC_{20} (95% *CL*) (mg/L)	χ^2 (*df*)	*P*
β-cypermethrin	Liaocanda 9	1.94 ± 0.22	0.0074 (0.0049-0.0098)	9.71 (13)	0.72

Table S2. RT-qPCR primer information of differentially expressed genes.

Gene	Direction	Primer Squence
CYP3A27	Forward	CTACCTGGATGCCTGCTT
	Reverse	TCTGGGTTTGGGAAGTAATCA
CYP3A56	Forward	TCGCAACAGTAGATGAAGT
	Reverse	ATGACCAGCATTGTAGCA
GSTD1	Forward	CTTCTCGTGGTGCTTTATTCG
	Reverse	TCGGCTAAGTAACAGGCAAT
CarE3	Forward	TGCCTGGAAGTGAAGATG
	Reverse	CAGATAACCGAGGGAACC
UGT2	Forward	CGTGTCCATCTTGTTTGTC
	Reverse	TTCGCAATGTGATAGCCT
ABCB1	Forward	CAAGGACCGCACTACGAT
	Reverse	CCACTCTCAGCCACAACT
ABCC4	Forward	AGAAGTATCGGAAGGTGGTT
	Reverse	CTGTATAAGGGTGTCGGTTTG
Hsp19.9	Forward	CTGCCGCTATTAGAGATGT
	Reverse	CCCTCAACCACGATGTAG
Hsp27	Forward	CAAGCAGGGAAACATTATGA
	Reverse	GCCAGAGATAGGTATCACTT
Hsp70	Forward	ATTCACCACCTACTCAGACA
	Reverse	CGTTCAGGATGCCGTTAG
Blm	Forward	CGCATACGCCATAGAGAAGA
	Reverse	ACTCAATCGGTTCCAATCTGT
Fancm	Forward	CGCTCACCTATACGACTC
	Reverse	TCTCTTTGCCGCATACAG
tubb1	Forward	CGTGATGGATGTGATTCG
	Reverse	TTGGAGATTAGGAGTGTACC

tubb4	Forward	GGACAGTTGAACGCAGAT
	Reverse	ACAAGCAGCCATCATATTCT
dnal1	Forward	TTGTTTGTCGGTAATCCACTCT
	Reverse	GCATGTCAGCAACTGTTAAGG
ATPsynbeta,	Forward	GCACCGCCCTTTATAGAA
	Reverse	CACCACCGAATAGACCAAT
SLC2A1	Forward	TTCGCAGGCTACATATTGAT
	Reverse	CTCGGCTATCGTCTGTTG
JHAMT	Forward	GCACCGAAGTTTAGATGGAAAG
	Reverse	CCACTCATATCACAACCAATTAGC
Gld	Forward	CTACTGGAACCACCTCAAGC
	Reverse	GGATTGTCACCAGCCTCTATTAG
far1	Forward	CGACTGCCCATTGTTATTC
	Reverse	ATAGCGACATCAACTGGAA
mei-41	Forward	TGGACTCGTTGGCTTTCA
	Reverse	CCATCACCCAGGGAATACAT
dna2	Forward	GTTGTCCCGCCCTGATAA
	Reverse	CGAACTGACTATGCTATTGCC
β-actin	Forward	CCAAAGGCCAACAGAGAGAAGA
	Reverse	CAAGAATGAGGGCTGGAAGAGA

Table S3. Transcriptome data statistic

Sample	Clean reads	Clean bases	≥Q30(%)	GC(%)	Mapped reads
CKX 1	47569834	7094376324	95.96	44.44	41829702 (87.93%)
CKX 2	48301256	7220059886	95.84	43.68	42088393 (87.14%)
CKX 3	52735822	7886468543	95.58	43.44	45427853 (86.14%)
TDX 1	51095088	7640756702	95.72	43.34	44234440 (86.57%)
TDX 2	49662478	7429612984	95.84	43.49	43008524 (86.60%)
TDX 3	51442304	7694022582	95.63	43.50	44617034 (86.73%)
